# Elasticity per Thickness Index for Breast Sonoelastography: Quantitative Analysis of Implant- and Clinically Related Peri-Implant Capsular Formation

**DOI:** 10.3390/diagnostics16213515

**Published:** 2026-10-27

**Authors:** Alicja Rzymska, Mikołaj Kubasik, Beata Pięta, Paweł Rzymski

**Affiliations:** 1 Student Scientific Society, Poznan University of Medical Sciences, 10 Fredry St., 61-701 Poznan, Poland; 2 Zlotowska Plastic Surgery Clinic, 63 Złotowska St., 60-184 Poznan, Poland; 3 Department of Practical Midwifery Education, Poznan University of Medical Sciences, 33 Polna St, 60-535 Poznan, Poland; 4 Department of Mother’s and Child’s Health and Minimally Invasive Gynecologic Surgery, Poznan University of Medical Sciences, 33 Polna St., 60-535 Poznan, Poland

**Keywords:** breast ultrasound, Sonoelastography, peri-implant capsule, capsular formation, capsular thickness, breast implants, quantitative imaging, elasticity-per-thickness index

## Abstract

**Background:** Peri-implant capsular formation remains a multifactorial process influenced by implant characteristics and patient-specific tissue responses. Ultrasound with shear-wave elastography offers evaluation of the biomechanical properties of both the capsule and surrounding breast tissues. Elastographic assessment does not distinguish intrinsic stiffness from the amount of fibrotic tissue. We introduced the Elasticity per Thickness Index (ETI) as an exploratory measure of capsular stiffness related to capsule thickness. **Methods**: This prospective study assessed peri-implant capsules and adjacent breast tissue compartments using B-mode ultrasound and shear-wave elastography over more than two years of follow-up. Capsular stiffness, thickness, and the exploratory Elasticity per Thickness Index (ETI; kPa/mm) were compared across implant characteristics, and their associations with clinical variables were analyzed. **Results**: Significant postoperative increases in stiffness were found across multiple native tissue compartments. Breast tissues remained on average stiffer than preoperatively throughout follow-up. Implant type or shape (F and M series, L3, MP EU, HP EU; anatomical vs. round) showed no association with stiffness changes, although round implants tended to yield higher fascial stiffness (*p* = 0.080). The ETI was independent of implant shape (*p* = 0.430). Higher-profile implants showed a nominally higher ETI than medium-profile implants (39.1 ± 13.4 vs. 31.9 ± 13.4 kPa/mm; unadjusted *p* = 0.050), which should be interpreted as an exploratory finding. The ETI was not significantly associated with implant profile after accounting for bilateral clustering (*p* = 0.455). Capsular stiffness was inversely associated with time from surgery to assessment (Rs = −0.329, *p* = 0.013), although this cross-sectional association does not demonstrate longitudinal softening. **Conclusions**: At the postoperative assessment, the peri-implant capsule showed a mean stiffness of 15.52 ± 3.81 kPa. The ETI may provide a complementary thickness-related description of capsular stiffness, although its reliability and clinical relevance require further validation. No robust association between implant profile and the ETI was identified after accounting for bilateral clustering.

## 1. Introduction

Breast implant design has undergone continuous refinement over recent decades in an effort to optimize biocompatibility and improve long-term integration with surrounding tissues. Despite these advances, the placement of any breast implant induces the formation of a peri-implant fibrous capsule as part of the foreign-body response, traditionally evaluated clinically using the Baker classification [1,2]. However, capsule morphology and biomechanical properties can also be assessed non-invasively with ultrasound, particularly shear-wave elastography, which provides quantitative information on tissue stiffness with high reproducibility.

Breast implants have undergone continuous technological evolution for many years, as manufacturers and clinicians strive to optimize biocompatibility and integration with surrounding tissue to reduce complications and improve clinical outcomes. This continuous improvement in implant design, including shell surface, fill material and mechanical properties, aims to achieve the most favorable tissue response and long-term performance. However, despite these advancements, every implanted foreign material inevitably induces fibrous capsule formation, which may vary in thickness and mechanical properties depending on implant features and host response. This peri-implant capsule is a normal tissue reaction, but in some cases it may become pathologically stiff and symptomatic [2,3]. Clinically, capsular contracture is most frequently classified using the Baker scale, a subjective clinical tool that grades firmness, shape distortion, and pain, yet this method has known limitations in objectivity. Because of these limitations, breast ultrasonography, especially when combined with elastography techniques like shear-wave elastography (SWE), has emerged as a more objective tool to assess capsule thickness and stiffness [4,5]. The prevalence of capsular contracture after breast implantation varies in the literature, with rates reported from approximately 5% to 25% in aesthetic and reconstructive settings, and although higher grades of contracture (Baker III–IV) often present symptoms such as firmness and distortion, many cases remain asymptomatic and do not necessitate surgical intervention [3,5].

Given the clinical importance of both implant design and diagnostic methodology, it remains critical to ascertain how implant features influence the biological process of peri-implant capsule formation and the mechanical behavior detected with advanced ultrasound imaging. Shear-wave elastography provides a quantitative estimate of tissue stiffness, whereas B-mode ultrasound enables measurement of capsule thickness. Because these two characteristics may convey complementary information, we introduced the Elasticity per Thickness Index (ETI), calculated as the ratio of capsular stiffness to capsule thickness. The ETI was used as exploratory arithmetic normalization intended to compare the measured stiffness-to-thickness relationship across capsules of different thicknesses. It was not assumed to represent a material property or to fully remove the influence of capsule thickness.

Previous studies indicate that capsule thickness and elastographic stiffness may both be associated with capsular fibrosis and contracture [5,6,7,8]. However, the functional form of the relationship between these variables, including whether it is linear, has not been established. The ETI was therefore evaluated as an exploratory stiffness-to-thickness ratio rather than as a validated correction for capsule thickness. Its purpose was to examine whether this combined parameter might provide information complementary to the separate reporting of stiffness and thickness. Therefore, the primary aim of this prospective observational study was to compare the stiffness of native breast tissue compartments before and after breast augmentation. We hypothesized that breast implantation would be associated with measurable changes in the elastographic properties of the surrounding tissues. The secondary aim was to explore whether postoperative capsular thickness, capsular stiffness, and the ETI were associated with implant characteristics, time from surgery to assessment, and selected clinical factors. Because the ETI had not previously been validated, analyses involving this ratio were considered exploratory and hypothesis-generating.

## 2. Materials and Methods

The study enrolled 35 consecutive women seeking elective breast augmentation at a single plastic surgery clinic, of whom 28 participants completed the full study protocol. Seven patients were excluded due to loss to follow-up. All patients received silicone-gel-filled implants from a single manufacturer (Eurosilicone, GC Aesthetics Cedex, France) to maintain homogeneity of implant characteristics. Most participants received anatomical implants (F2, F3, M2, M3, L3), whereas round implants (MP-EU) were used in 10 cases. Eurosilicone implants are manufactured in several height–projection combinations to accommodate different chest wall proportions. The height category determines the vertical dimension of the implant: full-height (F) implants provide the greatest upper-to-lower pole coverage, medium-height (M) implants offer a balanced vertical profile suitable for most anatomies, whereas low-height (L) implants are designed for patients with a shorter vertical breast footprint. Within each height group, the numerical suffix denotes the degree of projection, where “2” corresponds to a medium projection and “3” to a higher, more anterior projection. These combinations (e.g., F2, F3, M2, M3, L3) allow surgeons to tailor vertical height and forward projection independently, optimizing implant selection for chest wall morphology and desired breast contour. For the profile-based analysis, implants were classified according to projection rather than implant height. Anatomical implants with projection category 2 (F2 and M2) and round MP-EU implants were classified as medium-profile implants, whereas anatomical implants with projection category 3 (F3, M3, and L3) and round HP-EU implants were classified as high-profile implants.

All surgeries were performed by one experienced plastic surgeon using the standard dual-plane technique, as described in the previous literature, during the first 12 days of the menstrual cycle [9]. Patients were 21 to 40 years old (mean ± SD 31 ± 5 years). The Baker scale was evaluated clinically by the operating surgeon. Baker grade was assessed by the operating surgeon. No cases of Baker grade III–IV contracture occurred in the study cohort; therefore, all analyzed participants had Baker grade I–II. The study was approved by the Bioethics Committee of the Poznan University of Medical Sciences (approval no. 48/16). A study protocol was prepared before participant enrollment and received institutional ethics approval; however, the study was not prospectively registered in a public registry.

All ultrasound and elastographic assessments were obtained by an ultrasonographer with 15 years of experience using an Aixplorer system (SuperSonic Imagine, Aix-en-Provence, France), equipped with a 15 MHz linear-array transducer. Participants were examined in the supine position; measurements were obtained in radial imaging planes at the 3, 6, 9, and 12 o’clock positions, with the transducer resting lightly on the breast under its own weight and without additional manual compression. We used a 1 mm region of interest (ROI)—the minimum ROI supported by the equipment—placed directly at the tissue-implant interface. B-mode ultrasound was used to assess capsular thickness, whereas shear-wave elastography (SWE) provided quantitative measurements of tissue stiffness in the capsule, glandular tissue, fat, fascia, and muscle. The capsule was positioned centrally within the ROI under B-mode guidance to obtain the closest technically achievable approximation of capsular stiffness.

All measurements were performed in quadruplicate, and mean values were calculated. The examiner performing the elastographic measurements was blinded to the surgeon’s Baker grade assessment, while the surgeon assigning the Baker grade was blinded to the elastographic and tonometric results. Intra- and inter-observer reproducibility was not formally assessed. This prospective observational study assessed peri-implant capsules and adjacent breast tissue compartments using B-mode ultrasound and shear-wave elastography at a single postoperative follow-up assessment at 22–30 months. Additionally, the ETI was evaluated as an exploratory, thickness-related measure of capsular stiffness. No formal validation against histology, clinically significant capsular contracture, or repeated inter- and intra-observer measurements was performed.

We introduce the Elasticity per Thickness Index (ETI) as a parameter to explore elastographic stiffness values for capsule thickness. The ETI was calculated as the ratio of mean capsular stiffness measured by shear-wave elastography (kPa) to mean capsular thickness measured in B-mode ultrasound (mm), and expressed as kPa/mm.

$$\mathrm{ETI}=\frac{\mathrm{mean}\;\mathrm{capsular}\;\mathrm{stiffness}\;(\mathrm{kPa})}{\mathrm{mean}\;\mathrm{capsular}\;\mathrm{thickness}\;(\mathrm{mm})}$$

The ETI expresses the measured capsular stiffness relative to sonographically determined capsule thickness. It was used as exploratory normalization intended to facilitate comparisons between capsules of different thicknesses. Because the ETI has not been validated against histological composition, clinical capsular contracture, or independent biomechanical measurements, it should not be interpreted as a direct measure of material properties. This approach is conceptually analogous to classical biomechanical normalization of force to tissue geometry when assessing material properties. Thus, the ETI serves as a biomechanically grounded index reflecting the intensity of fibrosis in the context of tissue thickness, offering a more meaningful parameter for comparing peri-implant tissue quality across patients.

No formal a priori sample-size calculation was performed. The sample size was determined by the number of consecutive eligible participants who could be recruited and complete the study protocol during the study period. Therefore, the study should be regarded as exploratory, particularly with respect to comparisons across individual implant types. All results are presented as mean ± standard deviation (SD). The statistical software used was SigmaPlot v.13.0 (Systat, Palo Alto, CA, USA) and Python version 3.12.15 (Python Software Foundation, Wilmington, DE, USA), with the statsmodels package version 0.15.0 and SciPy version 1.13.1. Descriptive statistics were used for implant type distribution, surgical variables, and demographic characteristics. Continuous variables are presented as mean ± standard deviation. Normality was assessed using the Shapiro–Wilk test. Paired preoperative and postoperative measurements were compared using the paired Student’s *t*-test when the distribution of within-participant differences was approximately normal; otherwise, the Wilcoxon signed-rank test was used. Comparisons between two independent groups were performed using the independent-samples Student’s *t*-test for normally distributed variables and the Mann–Whitney U test otherwise. Comparisons across more than two implant-type groups were performed using one-way ANOVA or the Kruskal–Wallis test, depending on data distribution. Associations between continuous or ordinal variables were evaluated using Spearman’s rank correlation. All tests were two-sided, and *p* < 0.05 was considered statistically significant. Given the exploratory nature of the analyses, no adjustment for multiple comparisons was applied. The breast was used as the measurement unit, resulting in 56 breast-level observations from 28 women. The original exploratory analyses did not account for the correlation between bilateral measurements within the same participant; therefore, the resulting *p*-values should be interpreted cautiously and not as confirmatory evidence. The analyses were exploratory, and no adjustment for multiple comparisons was applied. Accordingly, *p*-values are reported as unadjusted, and findings close to the significance threshold should be interpreted cautiously. Each breast was considered an organ-level unit of measurement because local tissue responses to an implant may differ between breasts within the same participant, and postoperative events potentially affecting capsule formation may occur unilaterally. Because implant type and size were selected individually for each breast based on its specific clinical and anatomical characteristics, paired breasts did not necessarily receive identical implants; consequently, the breast was initially considered the primary unit of analysis. Nevertheless, bilateral observations share participant-level biological and clinical characteristics and are therefore not fully statistically independent. Accordingly, breast-level analyses were considered exploratory, and the absence of adjustment for within-participant clustering is acknowledged as a limitation. To account for the non-independence of bilateral observations, postoperative-minus-preoperative changes in tissue stiffness were additionally analyzed using linear mixed-effects models with participant as a random intercept. Bilateral agreement was evaluated by comparing dominant and non-dominant breasts and by calculating a two-way random-effects, absolute-agreement, single-measure intraclass correlation coefficient [ICC(A,1)]. As a sensitivity analysis, bilateral breast-level values were averaged within each participant and the mean participant-level change was tested against zero. Analyses were based on available cases; therefore, capsule thickness and ETI agreement analyses included 26 complete bilateral pairs, whereas capsular stiffness and tissue-change analyses included 28 pairs. All tests were two-sided. This study is reported in accordance with the Strengthening the Reporting of Observational Studies in Epidemiology (STROBE) guideline.

## 3. Results

No participant was excluded because of Baker grade III–IV capsular contracture or postoperative complications. We evaluated multiple ultrasound and elastographic features before surgery and after the follow-up period to capture all potential changes associated with peri-implant capsule formation. The statistical analysis revealed significant differences between preoperative and follow-up measurements in several tissue compartments, with the most pronounced increases observed in capsular stiffness. These data provide a foundation for exploring the relationships between tissue stiffness, thickness, and the Elasticity per Thickness Index (ETI) during the follow-up period. The statistical analysis and detailed differences of each feature are illustrated in Table 1. The Elasticity per Thickness Index (ETI), expressed as capsular stiffness to capsule thickness, showed a mean value of 35.76 ± 13.73 kPa/mm. Throughout follow-up, breast tissues remained on average stiffer than before surgery.

No statistically significant dependence of implant type (F2, F3, L3, M2, M3, MP EU, HP EU) on stiffness changes was observed across the evaluated breast tissues (Table 2).

Evaluation of the Elasticity to Thickness Index (ETI), reflecting stiffness per millimeter of peri-implant capsule thickness, revealed heterogeneity across implant types (Table 3). The ETI was nominally higher for high-profile than for medium-profile implants (39.074 ± 13.366 vs. 31.907 ± 13.382 kPa/mm), with a borderline unadjusted *p*-value of 0.050. Given the exploratory nature of the analysis and the absence of adjustment for multiple comparisons, this finding should not be considered confirmatory.

Significant positive correlations were observed between capsular thickness and the stiffness of all evaluated breast tissues (glandular, fat, muscle, and fascia), as presented in Table 4.

Follow-up time, age, parity, contraceptive use, sport activity, implant volume, and preoperative stiffness of glandular, fat, fascia, or muscle tissues showed no significant correlation with capsule thickness, stiffness or the ETI (Table 5). However, only a significant negative correlation was observed between follow-up duration and capsular stiffness (Rs = −0.329, *p* = 0.013).

After accounting for clustering of the two breasts within each participant, postoperative increases remained statistically significant for glandular, fat, muscle, and fascial stiffness (all *p* ≤ 0.025; Table 6). The participant-level sensitivity analysis based on the mean of both breasts yielded closely similar estimates and retained statistical significance for all four tissue compartments (all *p* ≤ 0.034). No systematic difference was detected between dominant and non-dominant breasts for capsule thickness, capsular stiffness, the ETI, or postoperative stiffness changes (all paired *p* ≥ 0.380). Bilateral absolute agreement was low for the ETI and tissue-change measures and low-to-fair for capsule thickness and capsular stiffness, indicating substantial within-participant, between-breast heterogeneity despite the absence of a directional side effect.

## 4. Discussion

Pathophysiology of Fibrosis

Fibrosis is characterized by increased deposition of the extracellular matrix, particularly collagen, which results in higher tissue stiffness compared with baseline [10]. Fibrotic healing generally involves increased collagen deposition, cross-linking, and fiber alignment, which collectively enhance tissue stiffness over months [11,12,13,14,15,16]. The proliferative phase, with an accumulation of extracellular matrix (ECM), lasts for three to six weeks, whereas subsequent remodeling lasts up to one year [17,18]. Postoperative fibrosis has been extensively described in the context of surgical adhesions. Experimental and clinical studies show that surgical trauma or injuries disrupt the mechanical homeostasis and trigger inflammation, fibroblast activation, and differentiation into myofibroblasts [19,20,21]. These processes lead to progressive collagen deposition and altered mechanical properties of the affected tissue [22]. Increased stiffness is a reproducible biomechanical feature of this response. Similar changes have been reported in musculoskeletal tissues. In a rat model of rotator cuff injury, muscle fiber bundle stiffness increased after tendon disruption and neuromuscular compromise [10]. This increase was associated with elevated collagen content at the tissue level. These findings indicate that fibrosis contributes directly to increased passive tissue stiffness. Although these mechanisms provide a plausible biological framework, they were not directly assessed in the present cohort and cannot be inferred solely from the elastographic findings.

Temporal changes in stiffness have also been documented in human tendon repair [23]. Shear-wave elastography studies after rotator cuff repair demonstrated a gradual increase in tendon stiffness during postoperative healing, first at the lateral footprint by 21% within the first 6 months, followed by progressive stiffening medially up to 12 months. However, tendon thickness decreased by 11% during the first 6 weeks, then stabilized, and tendons that were less stiff immediately postoperatively were more likely to be thinner at 6 weeks. The correlation between tendon stiffness and thickness was positive at 6 weeks postoperatively. These observations indicate that local material properties and spatial heterogeneity determine the timing and magnitude of biomechanical changes. Comparable mechanisms may occur in periprosthetic capsules, and the ETI introduced by us enables assessment of tissue stiffness independently of capsule thickness.

At the postoperative assessment, the capsule-centered peri-implant region showed a mean SWE stiffness of 15.52 ± 3.81 kPa. The ETI expresses capsular stiffness relative to capsule thickness and was evaluated as an exploratory composite ratio. This may help describe the relationship between these two measurements, but it does not establish whether higher stiffness results from capsule thickness, tissue composition, or other biomechanical factors. In particular, the ETI should not be interpreted as a thickness-independent measure of material stiffness [4,7,8,14,21].

Tissue stretching

Chronic mechanical tension may further contribute to the persistence of increased capsular stiffness over time. Prolonged mechanical stress maintains myofibroblasts in an activated state, increasing ECM protein and proteoglycan content and preventing the gradual relaxation of the tissue [21]. In peri-implant tissues, the implant creates a continuous tensional environment in which mechanical forces are transmitted to the forming capsule, promoting progressive tightening and hardening [4,21]. This tension-driven stabilization of the capsular matrix provides a plausible explanation for the sustained elevated stiffness observed in our long-term elastographic assessments. This mechanistic interpretation remains hypothetical because local tissue tension and mechanical loading were not directly measured in the present study.

Tendon as a tissue model

Recent mechanobiology research in tendon and ligament tissues provides additional insight relevant to the mechanical behavior of peri-implant capsules. Mechanical stretching has been shown to increase fiber density and promote collagen fiber thickening in tendons and ligaments, reflecting an adaptive response of fibroblastic cells to sustained tension [22,24]. Importantly, these findings question the traditional assumption that tissue strengthening is necessarily associated with increased stiffness. Dynamic mechanical properties may diverge from static measures, and standard ultrasound-based assessments often fail to capture these load-dependent biomechanical nuances [22]. In tendon and ligament healing, excessive or poorly regulated mechanostimulation promotes fibrosis, leading to adhesions that limit tissue gliding and weaken force transmission. Repaired tendon tissue often retains a higher proportion of type III relative to type I collagen, resulting in thinner fibrils and only partial recovery of native mechanical strength [22]. Similarly, human peri-implant capsules initially contain both collagen type III and type I, but the III:I ratio decreases during proliferation and maturation [25,26,27]. Experimental models further show that chronic mechanical stretch sustains myofibroblast activation through latent TGF-β1 signaling, reinforcing a pro-fibrotic environment [28,29]. Findings from tendon and ligament models may provide mechanistic context but cannot be directly extrapolated to the structurally and biologically distinct peri-implant capsule.

Molecular background

Connective tissue remodeling after injury or surgery is a dynamic process involving both quantitative and qualitative changes in the extracellular matrix (ECM). In pelvic organ prolapse, the ECM exhibits increased stiffness compared with non-prolapsed tissues, with altered collagen fiber orientation [20]. ECM stiffness itself regulates fibroblast activation and myofibroblast differentiation, creating a positive feedback loop that perpetuates fibrosis [12,13,21]. A recent study on breast implant capsules further shows that periostin enhances these processes [29].

Experimental and clinical studies further show that fibroblasts remodel their surrounding ECM in response to mechanical cues, aligning collagen fibers and modifying tissue mechanics to reach a mechanically stable state [14,24,30,31]. Similar mechanotransduction pathways operate in peri-implant capsules, where implant micromotion and persistent ECM tension can maintain myofibroblast activity and collagen cross-linking [32]. These mechanisms help explain why capsule stiffness may remain elevated even when structural measures such as thickness appear stable, which is consistent with the high Elasticity per Thickness Index (ETI) in our cohort. This suggests that both matrix composition and cellular remodeling contribute to the observed biomechanical properties independent of capsule thickness. This study introduces the ETI as a novel, thickness-related elastographic metric that captures capsular stiffness per millimeter of tissue—offering a level of biomechanical resolution not achievable with conventional SWE measurements alone.

In our cohort, capsular stiffness was inversely associated with the interval between surgery and the postoperative assessment. This observation is compatible with, but does not demonstrate, progressive mechanical relaxation or softening of the capsule. Because each participant underwent only one postoperative capsular assessment, the analysis represents a cross-sectional comparison between participants examined at different follow-up times. Serial within-participant measurements are required to establish the temporal trajectory of capsular stiffness and to determine whether the observed association reflects tissue remodeling or residual confounding. But this pattern is consistent with the expected transition from an early, tension-dependent fibrotic phase—dominated by sustained myofibroblast contraction and collagen cross-linking—to a later maturation phase characterized by reduced cellular activity, collagen realignment and progressive softening of the extracellular matrix. These molecular pathways were not measured in our study and are discussed only as potential mechanisms that may warrant investigation in relation to capsular elastographic properties.

Hormonal status

Although not directly assessed in our study, hormonal status represents a systemic variable that may subtly modulate ECM remodeling dynamics. Some findings indicate that lower estrogenic stimulation does not accelerate matrix turnover but instead dampens the normal remodeling machinery, resulting in a less dynamic ECM that is slower to reorganize and adapt [33]. Moreover, periprosthetic capsules have been shown to express estrogen receptors, suggesting that fibroblast activity within the capsule may be partially responsive to estrogen-dependent signaling [34]. Consequently, the approximately two-year physiological aging of participants in our study—during which a natural decline in estradiol could occur—might theoretically favor maintenance of an existing capsular structure rather than its softening, potentially modulating the elastographic changes measured over time. As circulating hormone concentrations and capsular receptor expression were not assessed, any contribution of hormonal signaling to the observed findings remains speculative. Previous work by Rzymski et al. evaluated shear-wave elastographic properties of normal breast tissue at different phases of the menstrual cycle, demonstrating temporal variation in tissue elasticity, although mean glandular and adipose tissue elasticity did not differ significantly across the cycle [35].

Elasticity per Thickness Index and biomechanics

The nominal profile-related difference observed in the original breast-level analysis was not confirmed after accounting for bilateral clustering. Accordingly, the present findings do not provide robust evidence that implant profile is associated with the ETI, and further mechanistic interpretation of this unconfirmed association would be premature. Implant profile (F2, F3, L3, M2, M3, MP EU, HP EU) height primarily reflects the anterior projection of the implant relative to the chest wall and therefore represents a mechanical challenge distinct from overall implant size. The impact of implant volume on peri-implant tissue response remains unclear in the literature [36]. However, our analysis showed no statistically significant association between implant volume and changes in tissue stiffness over long-term follow-up (Table 5). When implants were grouped by shape, no statistically significant differences in stiffness changes were observed between anatomical and round implants in glandular tissue (*p* = 0.337), fat (*p* = 0.275), or muscle (*p* = 0.435). However, in the fascial compartment, round implants were associated with higher stiffness values at follow-up compared with anatomical implants, with a near-significant difference (*p* = 0.080). No association was observed between implant height profile (medium or high) and changes in tissue stiffness in any of the analyzed compartments, but interestingly, its relationship with the ETI became apparent in the following analysis. High-profile implants were associated with higher stiffness per millimeter of capsule thickness compared with medium-profile implants, reaching borderline statistical significance (*p* = 0.050). These findings indicate that while implant type and shape do not significantly affect the ETI, implant profile height may influence the biomechanical quality of peri-implant fibrous tissue. The additional analyses accounting for bilateral clustering did not materially alter the principal findings, as postoperative increases in glandular, adipose, muscular, and fascial stiffness remained statistically significant in both mixed-effects models and participant-level sensitivity analyses. Although no systematic differences were observed between dominant and non-dominant breasts, the generally low bilateral agreement indicates substantial local variability in tissue response. These findings support retaining the breast as the organ-level unit of analysis while accounting for within-participant clustering. On the other hand, the nominal difference in the ETI between implant-profile groups observed in the original unadjusted analysis was not confirmed after accounting for bilateral clustering. Therefore, the present findings do not provide robust evidence of an association between implant profile and the ETI. The unadjusted result may reflect within-participant dependence, limited sample size, multiple comparisons, or random variation and should not be interpreted as evidence of a profile-related biomechanical effect.

A higher implant profile may induce greater and more focal tissue stretching, leading to increased mechanical loading of peri-implant soft tissues. In response, fibrous tissues undergo adaptive remodeling characterized by increased collagen deposition, altered fiber organization and increased stiffness per unit thickness, rather than simple thickening alone, as described above [37,38]. Such mechanical loading could potentially promote adaptive remodeling of the surrounding connective tissue, although this mechanism was not assessed in the present study. Larger or more projected breasts are exposed to higher mechanical demands, which may stimulate the formation of denser and stiffer fibrous structures. However, these tissues remain susceptible to age-related and hormonally driven degeneration, ultimately leading to stretching and ptosis over time [39,40].

Importantly, the ETI allows differentiation between capsules that are stiff due to increased thickness and those that exhibit elevated stiffness per millimeter, suggesting potential differences at the histological level. Experimental studies indicate that mechanically stretched connective tissues may exhibit reduced vascular perfusion, but stretch imposed by the implant may initially help maintain microvascular reactivity within the peri-implant capsule. But as the capsule thickens and stiffens, persistent mechanical stretch can contribute to endothelial dysfunction through mechanotransductive signaling and oxidative stress [41], thereby potentially altering vascular regulation and promoting fibrotic remodeling. Thus, the increased ETI values observed in high-profile implants may reflect qualitative changes in capsule microstructure rather than simply greater fibrous tissue accumulation. These vascular mechanisms provide a possible direction for future research but cannot explain the observed ETI differences on the basis of the present data.

The uniform, positive correlations between capsular stiffness (including the ETI) and the stiffness of glandular, fat, muscle and fascial tissues suggest that peri-implant fibrosis may reflect an underlying connective-tissue phenotype, rather than an isolated reaction local to the implant, a concept that aligns with the findings of Tiskratok et al. [14]. Importantly, this relationship persisted even after accounting for capsule thickness using the Elasticity per Thickness Index (ETI), indicating that capsular stiffness independent of tissue volume also correlates with the mechanical properties of surrounding breast tissues. Natural soft tissues share ECM-based structural determinants, particularly collagen content, fiber architecture, and cross-linking, which strongly influence mechanical behavior across organs. Other studies indicate that collagen composition and organization show systematic, tissue-specific patterns across ligaments, tendons and muscles [42,43], which support the idea that tissues within the same individual often share a global biomechanical profile. In our study, strong inter-tissue correlations were found within the native breast itself. These findings suggest that individuals with globally stiffer breast tissues, inherently denser or more collagen-rich connective tissue, may be more prone to both increased native tissue stiffness and the formation of a mechanically stronger and thicker contracture-prone peri-implant capsule.

Adipose tissue secretes adipokines such as leptin, which are necessary for normal muscle mass development and peak contractile tension, indicating a systemic influence of fat on muscle function beyond mechanical load alone [44]. Obesity is associated with greater absolute muscle strength as a chronic overload adaptation, although relative strength per body mass may be reduced [45,46].

Furthermore, in vivo data showed a positive trend between BMI and tendon stiffness in younger adults (*p* = 0.074) [47]. Taken together, these findings support a nuanced model in which adiposity exerts both mechanical and biochemical influences on musculoskeletal tissues, potentially contributing to global biomechanical phenotypes that include muscle, tendon, and connective tissue characteristics.

Pregnancy and breastfeeding induce long-term remodeling of breast connective tissues, including increased collagen deposition and altered fiber organization [48,49]. In our cohort, longer breastfeeding duration showed a positive trend with capsule stiffness (Rs = 0.248, *p* = 0.066), although it did not reach statistical significance, and no association was observed with the ETI, suggesting that capsular stiffness per millimeter is independent of lactation history and primarily reflects individual connective tissue phenotype. Age, parity, contraceptive use, sport activity, implant volume and preoperative stiffness of glandular, fat, fascia, or muscle tissues showed no significant correlations with thickness, stiffness, or the ETI.

### Limitations

The study has several limitations. The relatively small sample size and single-center design may limit the generalizability of the findings. The study included a heterogeneous range of implant types and profiles, resulting in relatively small subgroups and limiting the statistical power of comparisons between individual implant characteristics. Furthermore, the ETI is an exploratory ratio and has not yet been validated against histopathological findings, clinically significant Baker grades, or independent biomechanical measures. Inter- and intra-observer reproducibility of the ETI was also not specifically assessed. Therefore, the present findings should be regarded as hypothesis-generating. Moreover, calculation of the ETI as stiffness divided by thickness represents simple arithmetic normalization and implicitly treats their relationship as proportional. The present study did not establish that stiffness scales linearly with capsule thickness. The ETI therefore cannot be considered a validated thickness correction or a direct measure of material stiffness. An additional limitation is that bilateral measurements from the same participant were analyzed at the breast level without explicit adjustment for within-participant correlation. Thus, although 56 breasts were evaluated, the study included only 28 independent participants. Treating bilateral measurements as independent may have underestimated standard errors and produced overly optimistic *p*-values. Although treating each breast as an organ-level observation was biologically justified by the potential for unilateral local responses, this approach does not eliminate the statistical correlation between breasts from the same participant and may have resulted in underestimated standard errors. Because no Baker grade III–IV cases occurred, the findings cannot be generalized to clinically significant capsular contracture. The SWE ROI exceeded the sonographically measured capsule thickness in many examinations, which limits the tissue specificity of the measurements and may have introduced partial-volume contributions from adjacent tissues. Histologically, the peri-implant capsule represents a multilayered and spatially heterogeneous implant–tissue interface comprising cellular, stromal–vascular, and dense collagenous components; therefore, the thin echogenic line delineated on B-mode ultrasound may not reflect the full biological extent of the peri-implant tissue response. Consequently, although partial-volume contributions from adjacent tissues cannot be excluded, a capsule-centered SWE region of interest may still provide biologically relevant regional information and should be interpreted as reflecting the local mechanical environment of the implant–tissue interface rather than the intrinsic stiffness of the capsule alone. Larger, multicenter prospective studies with standardized imaging protocols and longer follow-up are needed to validate the ETI and further clarify its clinical relevance.

## 5. Conclusions

Breast augmentation resulted in a significant increase in tissue stiffness compared with preoperative values, and breast tissues remained on average stiffer throughout follow-up. Capsular stiffness was inversely associated with the interval from surgery to postoperative assessment, but the study design does not establish longitudinal softening or a temporal remodeling trajectory. Serial follow-up measurements are needed to clarify how capsular stiffness changes within individual patients. Implant shape was not associated with the ETI. The nominally higher ETI observed for high-profile implants was a borderline, unadjusted exploratory finding and should not be regarded as evidence of an established profile effect. A tendency toward higher stiffness was observed in women with longer breastfeeding, but did not reach statistical significance (Rs = 0.248, *p* = 0.066). The ETI provided an exploratory thickness-related description of capsular stiffness; however, its reproducibility, biological interpretation, and clinical validity require confirmation in dedicated studies. Multiple implant-related, tissue-related, and clinical associations were explored without multiplicity correction, increasing the possibility of chance findings. In particular, the profile-related ETI difference at an unadjusted *p*-value of 0.050 should be considered hypothesis-generating. Overall, these findings support a multifactorial process of postoperative remodeling, characterized by gradual mechanical recovery of native breast tissues despite persistent capsular formation and adaptation toward a new biomechanical steady state rather than progressive deformation proportional to implant volume.

## Figures and Tables

**Table 1:** Characteristics of ultrasound and elastographic features before surgery and after the follow-up period. Average stiffness of all tissues before and after implant surgery; values for capsule only after the follow-up period. All differences statistically significant in Student’s *t*-test for glandular stiffness, *p* < 0.001; fat stiffness, *p* = 0.001; fascia stiffness, *p* = 0.023; muscle stiffness, *p* < 0.001. Preoperative and postoperative measurements of native breast tissues were obtained from the same breasts and were compared using the paired Student’s *t*-test. Changes were calculated as postoperative minus preoperative values.

Ultrasound and Elastographic Features	Mean	SD	Min	Max
Average capsule thickness (mm)	0.471	0.177	0.2	1.3
Follow-up time (months)	24.3	3.2	22	30
Mean of average glandular stiffness (kPa) after follow-up	10.167	3.051	4.225	17.35
Mean of average glandular stiffness (kPa) before surgery	7.69	2.419	3.775	14.35
Mean of average fat stiffness (kPa) after follow-up	8.749	3.17	4.45	16.55
Mean of average fat stiffness (kPa) before surgery	6.715	2.696	2.05	15.1
Mean of average fascia stiffness (kPa) after follow-up	12.221	3.51	4.65	21.25
Mean of average fascia stiffness (kPa) before surgery	10.56	4.752	4.5	29.9
Mean of average muscle stiffness (kPa) after follow-up	12.979	4.215	5	24.4
Mean of average muscle stiffness (kPa) before surgery	9.938	4.269	2.1	26.55
Mean of average capsular stiffness (kPa) after follow-up	15.523	3.809	6.875	25.425
Elasticity per Thickness Index of capsule (kPa/mm)	35.756	13.729	14.731	79.625
	Mean change (95%CI)			
Change in glandular stiffness (kPa)	2.48 (1.44–3.51)	3.872	−4.525	12.725
Change in fat stiffness (kPa)	2.03 (0.84 to 3.22)	4.441	−8.325	12.85
Change in muscle stiffness (kPa)	3.04 (1.39 to 4.70)	6.178	−15.45	14.9
Change in fascia stiffness (kPa)	1.66 (0.24 to 3.09)	5.328	−10.95	11

**Table 2:** Change in breast tissue stiffness (kPa) evaluated in elastography dependent on implant type.

	Glandular Stiffness Change		Fat Stiffness Change		Muscle Stiffness Change		Fascia Stiffness Change	
Implant Type	Mean (kPa)	SD	Mean (kPa)	SD	Mean (kPa)	SD	Mean (kPa)	SD
F2 (*n* = 4)	0.6	1.8	3.0	2.0	−1.0	4.7	−2.4	2.6
F3 (*n* = 4)	0.1	1.0	−0.6	3.6	2.5	12.6	0.1	6.4
L3 (*n* = 6)	0.4	2.2	0.3	5.9	−0.2	2.8	−1.5	3.1
M2 (*n* = 15)	1.4	1.5	1.4	3.6	1.8	5.4	1.3	6.6
M3 (*n* = 17)	1.5	2.3	2.8	5.5	5.5	5.6	2.9	5.0
MP EU (*n* = 2)	1.6	1.9	3.0	3.8	4.0	6.7	4.9	4.0
HP EU (*n* = 8)	2.5	2.6	5.3	3.0	6.4	1.7	2.1	0.7
	One-way ANOVA *p* = 0.636		One-way ANOVA *p* = 0.607		One-way ANOVA *p* = 0.276		One-way ANOVA *p* = 0.189	
**Anatomical vs. Round**								
Anatomical (*n* = 46)	1.1	1.9	1.7	4.6	2.7	6.2	1.1	5.5
Round (*n* = 10)	1.8	1.9	3.4	3.6	4.4	6.0	4.3	3.7
	Student’s *t-*test *p* = 0.337		Student’s *t-*test *p* = 0.275		Student’s *t-*test *p* = 0.435		Student’s *t-*test *p* = 0.080	
**Implant profile**								
Medium (*n* = 21)	1.3	1.6	2.1	3.5	2.0	5.7	1.8	5.9
High (*n* = 35)	1.1	2.2	2.0	5.3	4.0	6.0	1.5	4.9
	Student’s *t-*test *p* = 0.702		Student’s *t-*test *p* = 0.875		Student’s *t-*test *p* = 0.235		Student’s *t-*test *p* = 0.845	

n—number of breasts with implant for analysis.

**Table 3:** ETI (kPa/mm) values of peri-implant capsule and the influence of implant types and features. None of the comparisons met the prespecified significance threshold of *p* < 0.05. The profile-related difference at *p* = 0.050 is reported as a borderline, exploratory finding.

Implant Type	Mean (kPa/mm)	SD	
F2	28.256	7.442	*p* = 0.099 Anova on Ranks (Kruskal–Wallis test)
F3	42.304	10.388	
L3	35.185	9.999	
M2	31.647	15.634	
M3	41.977	13.982	
MP EU	34.514	11.921	
HP EU	19.603	4.058	
**Anatomical vs. Round**			
Anatomical	36.667	13.94	Student’s *t-t*est *p* = 0.430
Round	31.2	12.324	
**Implant profile**			
Medium	31.907	13.382	Student’s *t-t*est *p* = 0.050
High	39.074	13.366	

**Table 4:** Correlations between ETI (kPa/mm) values of peri-implant capsule and mean stiffness changes [kPa] in other breast tissues after surgery (follow-up). Statistically significant values are highlighted in bold. Avg—average.

	Capsule ETI (kPa/mm)	Glandular Stiffness Change (kPa)	Fat Stiffness Change (kPa)	Muscle Stiffness Change (kPa)	Fascia Stiffness (kPa) at Postoperative Assessment
Capsular Avg thickness (mm)	**Rs = 0.576** ***p* < 0.001**	**Rs = 0.566** ***p* < 0.001**	**Rs = 0.641** ***p* < 0.001**	**Rs = 0.575** ***p* < 0.001**	**Rs = 0.291** ***p* = 0.02**
Capsule ETI (kPa/mm)		**Rs = 0.342** ***p* = 0.011**	**Rs = 0.319** ***p* = 0.019**	**Rs = 0.419** ***p* = 0.001**	**Rs = 0.466** ***p* < 0.001**
Glandular stiffness change (kPa)			**Rs = 0.822** ***p* < 0.001**	**Rs = 0.478** ***p* < 0.001**	**Rs = 0.396** ***p* = 0.002**
Fat stiffness change (kPa)				**Rs = 0.462** ***p* < 0.001**	**Rs = 0.437** ***p* < 0.001**
Muscle stiffness change (kPa)					**Rs = 0.709** ***p* < 0.001**

**Table 5:** Correlations of capsule thickness, capsular stiffness, and ETI at postoperative assessment with clinical and implant-related variables. Statistically significant Rs and *p*-values highlighted. Avg—average. Statistically significant values are highlighted in bold.

	Mean Capsule Thickness (mm)	Capsule Avg Stiffness at Postoperative Assessment (kPa)	ETI (kPa/mm)
Age (years)	Rs = 0.185	Rs = 0.187	Rs = −0.0606
	*p* = 0.172	*p* = 0.168	*p* = 0.662
Deliveries (*n*)	Rs = 0.0594	Rs = 0.161	Rs = 0.0938
	*p* = 0.662	*p* = 0.235	*p* = 0.498
Breastfeeding (months)	Rs = 0.208	Rs = 0.248	Rs = 0.0317
	*p* = 0.124	*p* = 0.0659	*p* = 0.819
Use of contraceptives (months)	Rs = −0.0624	Rs = −0.0418	Rs = −0.0711
	*p* = 0.653	*p* = 0.763	*p* = 0.615
Sport (hours/week)	Rs = 0.0972	Rs = 0.0211	Rs = −0.0961
	*p* = 0.475	*p* = 0.877	*p* = 0.488
Implant volume (cm^3^)	Rs = 0.00381	Rs = 0.199	Rs = 0.159
	*p* = 0.977	*p* = 0.141	*p* = 0.249
Follow-up time (months)	Rs = −0.172	**Rs = −0.329**	Rs = −0.121
	*p* = 0.204	***p* = 0.0135**	*p* = 0.382
Glandular tissue Avg stiffness before surgery (kPa)	Rs = 0.105	Rs = −0.0735	Rs = −0.168
	*p* = 0.438	*p* = 0.589	*p* = 0.223
Fat tissue Avg stiffness before surgery (kPa)	Rs = 0.193	Rs = −0.157	Rs = −0.115
	*p* = 0.153	*p* = 0.246	*p* = 0.405
Fascia Avg stiffness before surgery (kPa)	Rs = −0.096	Rs = 0.128	Rs = −0.0981
	*p* = 0.478	*p* = 0.347	*p* = 0.479
Muscle tissue Avg stiffness before surgery (kPa)	Rs = 0.031	Rs = −0.0867	Rs = −0.169
	*p* = 0.815	*p* = 0.524	*p* = 0.22

**Table 6:** Mixed-effects, bilateral agreement, and participant-level sensitivity analyses. Statistically significant values are highlighted in bold.

Outcome	Mixed-Effects Estimate (95% CI); *p*-Value	Sensitivity Estimate (95% CI); *p*-Value	Dominant Minus Non-Dominant Breast (95% CI); *p*-Value	ICC(A,1)
Glandular stiffness change (kPa)	1.24 (0.71 to 1.77); ***p* < 0.001**	1.24 (0.69 to 1.79); ***p* < 0.001**	0.28 (−0.75 to 1.31); *p* = 0.582	0.067
Fat stiffness change (kPa)	2.03 (0.87 to 3.20); ***p* < 0.001**	2.03 (0.87 to 3.20); ***p* = 0.001**	0.03 (−2.56 to 2.61); *p* = 0.982	−0.110
Muscle stiffness change (kPa)	3.04 (1.25 to 4.83); ***p* < 0.001**	3.04 (1.17 to 4.91); ***p* = 0.003**	−1.31 (−4.32 to 1.70); *p* = 0.380	0.217
Fascial stiffness change (kPa)	1.66 (0.21 to 3.12); ***p* = 0.025**	1.66 (0.14 to 3.18); ***p* = 0.034**	0.23 (−2.62 to 3.08); *p* = 0.869	0.069
Capsule thickness (mm)	Not applicable	Not applicable	0.04 (−0.06 to 0.14); *p* = 0.392	0.153
Capsular stiffness (kPa)	Not applicable	Not applicable	−0.18 (−2.02 to 1.65); *p* = 0.839	0.249
ETI (kPa/mm)	Not applicable	Not applicable	−1.50 (−9.33 to 6.33); *p* = 0.697	0.042

Notes: Mixed-effects estimates are postoperative minus preoperative mean changes from linear mixed-effects models with participant as a random intercept (56 breasts nested within 28 participants). Sensitivity estimates are based on participant-level bilateral means (*n* = 28). Bilateral differences are dominant minus non-dominant breast. ICC(A,1) denotes a two-way random-effects, absolute-agreement, single-measure intraclass correlation coefficient. Capsule thickness and ETI agreement analyses included 26 complete bilateral pairs because two breast-level values were unavailable. “Not applicable” indicates that a change-from-zero analysis was not clinically meaningful for single postoperative capsule measurements.

## Data Availability

The datasets presented in this article are not readily available because because of privacy. Requests to access the datasets should be directed to Pawel Rzymski.

## Abbreviations

The following abbreviations are used in this manuscript:

ETI	Elasticity per thickness index
SWE	Shear-wave elastography
SE	Strain elastography
ROI	Region of interest
ECM	Extracellular matrix
BMI	Body mass index
